# Investigating Symptomatic Vitreous Opacities: An Online Survey and Field of View Reconstruction

**DOI:** 10.1055/a-2676-7455

**Published:** 2025-10-15

**Authors:** Jaryi Lippek, Leonie Rynko, Carsten Framme, Omid Kermani, Sonja Johannsmeier, Tammo Ripken

**Affiliations:** 1 Life Sciences, Laser Zentrum Hannover e. V., Hannover, Germany; 2 Communication, Laser Zentrum Hannover e. V., Hannover, Germany; 3 University Eye Hospital, Hannover Medical School, Hannover, Germany; 4 Augen- und Laserklinik Köln, Artemis Augenklinik am Neumarkt, Köln, Germany; 5 Laser Zentrum Hannover e. V., Lower Saxony Center for Biomedical Engineering, Implant Research and Development (NIFE), Hannover, Germany

**Keywords:** vitreous opacities, vitreous floaters, survey, myopia, field of view, quality of life, Glaskörper, Glaskörpertrübung, Floater, Kurzsichtigkeit, Umfrage, Sichtfeld

## Abstract

**Background** Symptomatic vitreous opacities (SVO) are a condition that can significantly affect patientsʼ quality of life. This online survey aims to contribute to the scientific understanding of symptomatic vitreous opacities, by investigating the subjective burden and individual perception of floaters, using a novel tool for field of view reconstruction. In addition, correlations with demographic data and refractive status will be analysed to identify patterns and potential influencing factors.

**Methods** We introduce a VO-related online survey covering patient education, experience with floaters and treatment, refraction error and eye diseases, quality of life, and a new sketching tool to simulate the individual field of view with VO. The data were collected between August 2022 and November 2023 and are analysed for correlations; participantsʼ answers are compared to the sketching tool results. We used linear regression with gradient descent to test whether a subjective suffering index from the questionnaire can be matched with an objective suffering index from the new sketching tool.

**Results** A total of 1502 people participated in the survey, of whom, 1384 reconstructed their field of view by positioning a total of 10,571 VO. There was no robust correlation between the sketched floaters and the subjective degree of suffering. Translucent VO types are more frequent in younger attendees. The dataset shows a higher prevalence of myopic participants and a lower prevalence of hyperopes with VO compared to global data.

**Conclusion** We were able to collect the largest dataset to date quantifying individual experience with floaters. As evidenced by the large number of participants with SVO and individual remarks on their psychological burden, greater effort is needed to improve the care for floater patients.

## Zusammenfassung

**Hintergrund** Symptomatische Glaskörpertrübungen können die Lebensqualität der Betroffenen erheblich beeinträchtigen. Diese Online-Umfrage soll zum wissenschaftlichen Verständnis symptomatischer Glaskörpertrübungen beitragen, indem die subjektive Belastung und die individuelle Wahrnehmung von Floatern mithilfe eines neuartigen Tools zur Rekonstruktion des Gesichtsfeldes ermittelt werden. Darüber hinaus werden Korrelationen zu demografischen Daten und dem Refraktionsstatus analysiert, um potenzielle Einflussfaktoren zu identifizieren.

**Methoden** Die hier präsentierte Online-Umfrage zum Thema Glaskörpertrübung umfasst Zusammenhänge zu Patientenaufklärung, Erfahrungen mit Floatern und Behandlung, Refraktionsfehlern und Augenerkrankungen, Lebensqualität und einem neuen Skizzierwerkzeug zur Simulation des individuellen Gesichtsfeldes mit VO. Die zwischen August 2022 und November 2023 gesammelten Daten wurden auf Korrelationen untersucht und die Antworten der Teilnehmer mit den Ergebnissen des Skizzierwerkzeug verglichen. Wir haben eine lineare Regression mit Gradientenabstieg verwendet, um zu prüfen, ob ein subjektiver Leidensindex aus dem Fragebogen mit einem objektiven Leidensindex aus dem neuen Skizzierwerkzeug übereinstimmen kann.

**Ergebnisse** Insgesamt nahmen 1502 Personen an der Umfrage teil, von denen 1384 ihr Sichtfeld durch Positionierung von insgesamt 10 571 Floater rekonstruierten. Es gab keine robuste Korrelation zwischen den skizzierten Floatern und dem subjektiven Leidensdruck. Transluzente Trübungen treten häufiger bei jüngeren Teilnehmern auf. Der Datensatz zeigt eine höhere Prävalenz von myopen und eine geringere Prävalenz von hyperopen Teilnehmern mit Floatern im Vergleich zu globalen Daten.

**Schlussfolgerung** Wir konnten den bisher größten uns bekannten Datensatz zur Quantifizierung individueller Erfahrungen mit Glaskörpertrübungen erheben. Wie die große Zahl der Teilnehmer mit symptomatischen Glaskörpertrübungen und die individuellen Aussagen zur psychischen Belastung zeigen, sind weitere Anstrengungen notwendig, um die Versorgung von Floater-Patienten zu verbessern.

## List of Abbreviations

BT: Blur and transparancy
DoC: Degree of coverage
DV: Disturbance value
GDLR: gradient descent for linear regression
LV: Laser vitreolysis
OSI: Objective Suffering Index
Pos: Position
PPV: Pars planar vitrectomy
PVD: Posterior vitreous detachment
SSI: Subjective Suffering Index
SVO: Symptomatic vitreous opacities
VO: Vitreous opacities
 

## Introduction

Vitreous opacities (VO) are a common phenomenon especially in older people. They consist of inhomogeneities in the vitreous body, which naturally consists of about 98 – 99% water and a network of collagen fibrils stabilized by hyaluronan and proteoglycans 1 , 2 , 3 , 4 . Imbalances in this network result in the formation of optically dense areas, which lead to the perception of primary vitreous floaters. Whereby the floater is the visual phenomenon of scattered light caused by the VO, which consists of vitreous-own material 5 . Their occurrence is often associated with posterior vitreous detachment (PVD) and increasingly with axial myopia, which can also occur in young people 6 , 7 , 8 , 9 . VO cause light scattering and shadowing on the retina, which is perceived as clouds, threads or dots that may float with the eye movement 10 , 11 . Secondary vitreous floaters are commonly caused by material exogenous to the vitreous, like cells, proteins, and blood from hemorrhages 5 .

For most people, floaters are harmless and tolerable. Patients with persistent symptomatic vitreous opacities (SVO) however can experience a significant impact on their quality of life 12 . The constant disturbance in the field of vision leads to anxiety, stress, and depression 13 , 14 , 15 , 16 . Additionally, contrast sensitivity has been shown to be reduced in floater patients 17 , 18 . This has a negative effect on daily tasks and work performance as well as social life 19 . Non-invasive laser vitreolysis (LV) and surgical pars planar vitrectomy (PPV) are available as treatments. However, cataract formation is common in the years following PPV, making it unattractive for young phakic patients 20 , 21 . Nevertheless, there have been improvements in recent years due to the use of smaller instruments 22 . The variable success rates of LV give the impression that the technology could close the gap between conservative therapy and vitrectomy, but is not yet technically mature 5 , 23 24 25 26 .

Determining individual patient suffering and treatment expectations can be challenging for physicians. 2022 Senra et al. reviewed five studies that investigated the psychological effects of floaters and found that the topic is still very unexplored. The heterogeneity of the study design, the small number of participants and the lack of control groups make it difficult to draw general conclusions 12 .

Here, we present a comprehensive online survey to quantify personal experiences and suffering, treatment success and possible causes. The presented data includes 1502 participants, making it the largest scientific survey conducted on floaters to date, to our knowledge. Further, we introduce a new sketching tool that allows individuals to depict their field of vision with respect to their floaters.

## Methods

### Data collection

The survey was launched on the website of Laser Zentrum Hannover e.V. in August 2022. It was made generally accessible in German and English and was promoted on social media. Therefore, it is not comparable to the standard of a clinical trial. No compensation was given to participants. This article uses the dataset collected until November 2023. Nine people were removed from the set due to highly inconsistent responses, resulting in 1502 entries. All entries are anonymous, and data collection was performed in accordance with EUʼs General Data Protection Regulation.

Data analysis and plotting was done in JupyterLab with the python libraries matplotlib, pandas and seaborn.

We categorized the survey into following subsections:

- Patient education. Causes and treatment options for floaters believed to be accurate by participants to investigate the extent to which pseudoscientific explanations are believed by affected people.
- Personal experience. Duration and severity of seeing floaters, types of floaters and possible correlations with specific events such as trauma or disease. The sketching tool (see below) was included here.
- Quality of life. Degree of subjective association of floaters with a decreased quality of life, such as not pursuing hobbies, not participating in public life, difficulties at work or while driving.
- Treatment. Number of doctors consulted and their response, treatment(s) performed and perceived success and adverse effects to quantify the personal experience and assess the quality of available treatment.
- Quality of vision and health. General vision, pre-existing conditions, operations and their correlation with the occurrence of floaters, overall health as well as available patient care. In the results, we focus on the influence of refractive error on floaters.

### Floater sketcher

**Fig. 1:**
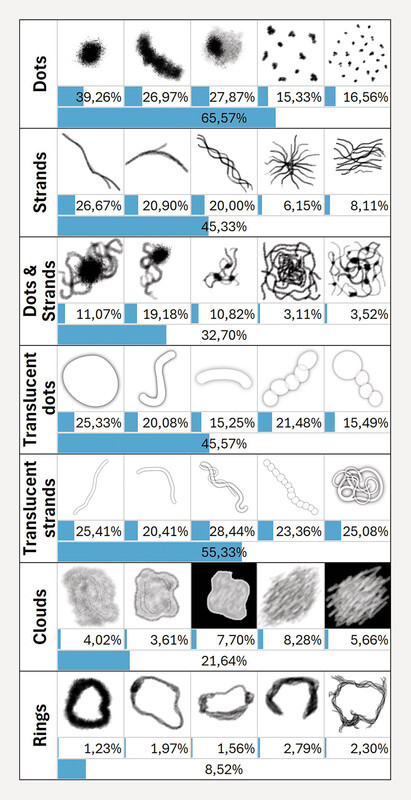
Floater classes of the sketcher tool. Percentages indicate how many attendees used that type or class of floaters at least once.

The floater sketcher was implemented into the survey. Participants were asked to position their floaters on a circular surface representing the right or left eyeʼs view. A set of 35 floaters in seven categories was given to choose from ( Fig. 1 ), based on patient reports and literature 27 , 28 .

The floaters could be scaled in size, rotated, repositioned, and adjusted with respect to transparency and blur. Each step performed by the participants was stored in a database for each floater and linked to their respective questionnaire.

### Subjective Suffering Index (SSI)

The 17 answers to six matrix questions were scored. The sum provides a total of 70 possible values and was used as the subjective suffering index (see SSI questions and scoring in the supplementary material Table S1).

### Objective Suffering Index (OSI)

Using the sketching tool, we attempted to provide a more objective means of quantifying personal suffering. This index was calculated from five parameters: degree of coverage, position, blur, transparency and number of floaters. The parameters where then individually weighed by a factor x _i_ to achieve maximum correlation with the corresponding SSI. First, we determined a disturbance value (DV) for each individual floater based on the first four parameters:

#### Degree of coverage (DoC)

Assuming that larger floaters would be more bothersome, we calculated the percentage of floater pixels covering the sketching area and scaled it from 0 to 1. This included white space in the bounding box (see Fig. 1 ), which is the same size for all floaters.

#### Position (Pos)

As object perception takes place in the central vision, we assumed that a floater is most disturbing in the center of each eyeʼs field of view. A Pos value of one was assigned for such floaters, decreasing exponentially towards the periphery. Due to additional complexity for the technical implementation of the drawing tool and for user operation, we did not take the movement of floaters into account. Even though this would have affected the DV of each individual floater.

#### Blur and transparency (BT)

The blur effect in the floater sketcher is a gaussian blur adjustable in ten steps. Transparency changes the alpha value of each pixel, also adjustable in ten steps.

A floater with the lowest respective settings was assigned the highest BT value, and highly transparent floaters were assigned low values. Since blur and transparency affect the perception of various floater types differently and not independently of each other, the resulting overall alpha value extracted from sketch histograms was used as a proxy for the combined effect. The two parameters were combined into a single value to allow for comparison across the different floater types.

#### Disturbance Value for one floater

The DV _i_ is calculated for each individual floater i by summing up the parameters DoC _i_ , Pos _i_ and BT _i_ . As each parameter has a value between 0 and 1, we divide by three for mapping.

${DV}_{i}={DoC}_{1}+{Pos}_{i}+{BT}_{i}/3$

The floater with the highest value DV _max_ is perceived as most disturbing for the person based on the previous assumptions.

#### OSI-Calculation for one person

A sum parameter for all floaters of an individuum was calculated as the mean of the respective parameter values plus the respective value of the DV _max_ floater (equation 2–4). This ensured that the influence of the most disturbing floater is not diluted by many less bothersome floaters.

${DoC}_{sum}=\;\left(\frac{1}{n}\right)\times \sum_{i=1}^{n}{DoC}_{i}+\;{DoC}_{{DV}_{max}}$

${Pos}_{sum}=\;\left(\frac{1}{n}\right)\times \sum_{i=1}^{n}Pos_{i}+\;Pos_{{DV}_{max}}$

$BT_{sum}=\;\left(\frac{1}{n}\right)\times \sum_{i=1}^{n}BT_{i}+\;BT_{{DV}_{max}}$

For the personʼs OSI from the sketcher tool, the parameters DoC _sum_ , Pos _sum_ , BT _sum_ and the total number n of floaters need to be weighted with a factor x _i_ . We employed a gradient descent for linear regression (GDLR) to iteratively determine the respective factors to match the result to the SSI determined from the questions, with x _0_ as offset:

$SSI=x_{0}+x_{1}\times Size_{sum}+x_{2}\times {Pos}_{sum}+x_{3}\times {BT}_{sum}+x_{4}\times n$

The resulting x _i_ values were multiplied with their respective parameter (e.g. Pos _sum_ ) and total number of floaters of that person.

## Results

This paper focusses on presenting the most important outcomes of the survey. The entire dataset will be made available upon request. Of all participants, 69.1% (1038) were male, 30.6% (459) were female, and five individuals identified as diverse. Their age ranged from 11 to 86 years (mean 35 years), whereby 81% were between 18 and 45 years old. 41% were in their twenties. The dataset includes individuals from 99 different countries of origin, with the majority living in China (22.8%), Germany (21.8%) and the United States (11.0%), followed by the United Kingdom (3.4%). 97.8% of the participants were seeing floaters.

### Patient education

Most of the respondents (63.6%) attribute the development of floaters to aging, and 50.1% believe it is associated with nearsightedness. Only 11.4% are uncertain about any possible causes.

**Fig. 2:**
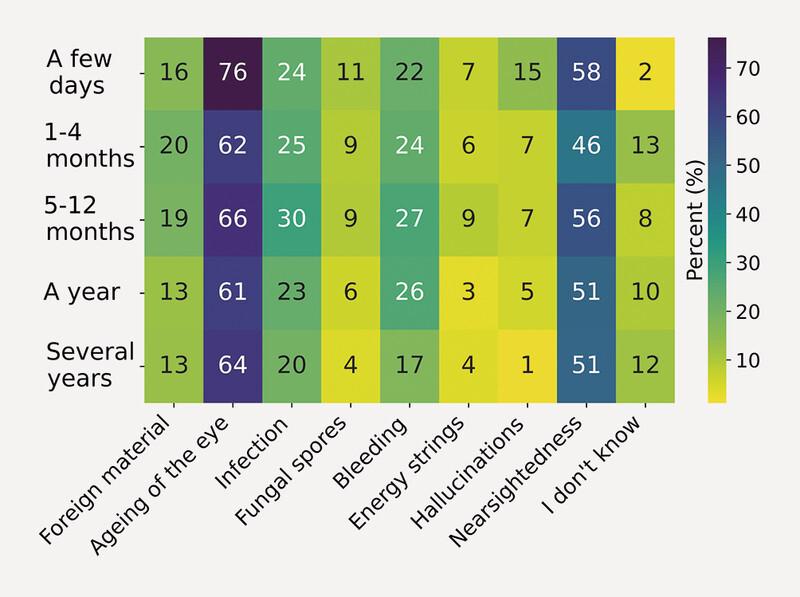
Duration of seeing floaters vs. believed causes of floaters. Believes in scientific explanations for floaters like aging and myopia are consistently high, while pseudoscientific explanations like fungus, energy strings and hallucinations become less important over the time.

Upon correlation with the question “How long have you been seeing floaters?”, it becomes evident that alternative or misleading explanations become less popular over time ( Fig. 2 ).

These findings suggest that affected people tend to converge towards a more accurate understanding of the potential causes of vitreous opacities.

A comparable trend was not observed for possible treatments of floaters. Laser vitreolysis and vitrectomy were well known (65.4% and 62.4%, respectively), while 9.0% chose meditation and 7.5% chose acoustic frequencies.

### Personal experience and quality of life

**Fig. 3:**
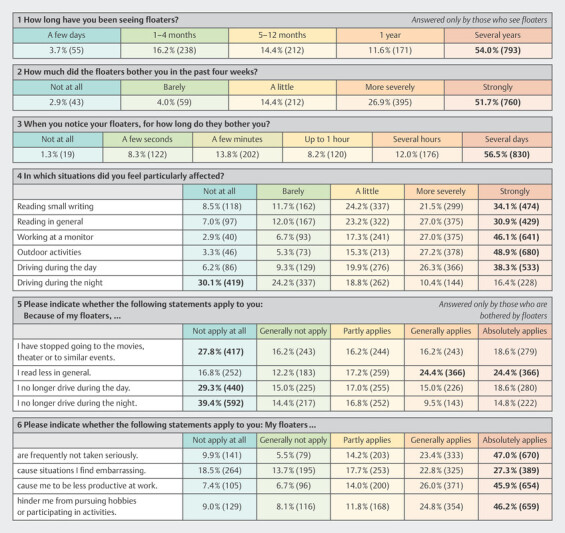
Questions related to personal floater experience and quality of life. Max. value marked bold.

Fig. 3 summarizes how floaters affect the participantsʼ everyday life. Most participants felt strongly affected during all activities available for selection, except driving at night. Regarding social activities like movies and theater visits, only 27.8% reported that floaters did not hinder their participation ( Fig. 3 , section 5). 47.0% absolutely agreed with the statement that their floaters are not taken seriously by others ( Fig. 3 , section 6).

Most individuals could not name a connection between floater occurrence and a specific event. Surprisingly, 32.1% (472) of participants associated their floaters with flashes of light, a phenomenon described by people with PVD, a potential cause of floaters 29 . However, only 7.0% (103) associated their floaters with PVD, 64 of whom in turn reported seeing flashes.

### Floater sketcher

Fig. 1 shows the set of floaters available in the Floater sketcher. A total of 10,571 floaters were positioned. Dot- and string-like floaters were used predominantly. Examples from the survey data can be found in the supplementary material Fig. S1.

**Fig. 4:**
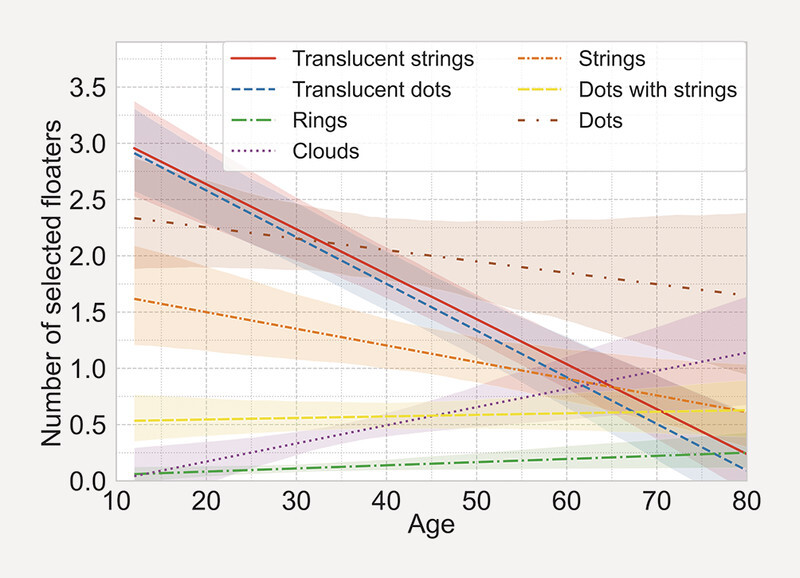
Regression of average number of selected floater class vs. age. Confidence Interval: 95% Younger individuals used translucent floaters more frequently. Solid dots and strings are present over all ages. Cloud like floater seems to appear more often in older eyes.

Fig. 4 shows the average number of times a floater class was selected with the corresponding age of the participants. The data shows that with increasing age the translucent floaters were used less frequently. A slight increase in cloud floaters is noticeably with older age. There is also a trend of decreasing numbers of dot and strand floaters with age, which is however relativized by the confidence intervals.

1384 participants used the sketching tool, 83.4% of which rated its usage as sufficiently to very good, and 46.7% as good to very good. The input from participants who rated the tool as less then sufficiently good was excluded from OSI calculations.

OSI (from sketching tool) and SSI (from questionnaire) show a weak positive linear correlation (R = 0.325). The histogram of the deviations of the calculated OSI from the SSI roughly follows a gaussian shape, with an average of 0.46 percentage points deviation. The standard deviation is 21.8%, with 83.4% of all values lying within one standard deviation from the mean. For 56.6% of the participants, the OSI deviates only 5 percentage points from their SSI value. However, the variability within the deviations was very large, ranging from 0 to 60 percentage points. The correlation is therefore not robust enough to make generally valid assumptions concerning a specific individual, but demonstrates the general possibility of describing the subjective suffering with an objective tool.

### Treatment

**Fig. 5:**
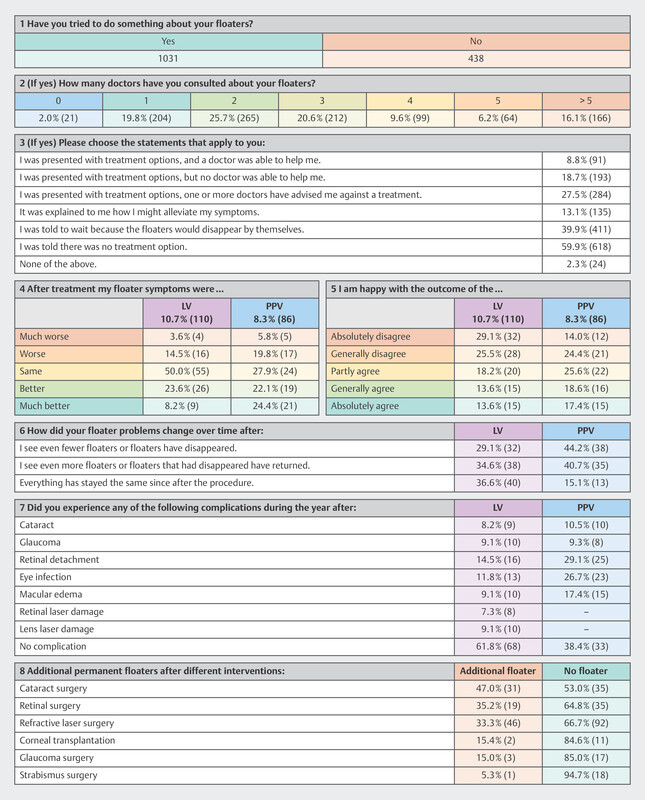
Survey results to treatment search, procedure and outcome.

Approximately two thirds of the participants have sought professional help ( Fig. 5 ), more than 78.2% of which visited two or more doctors ( Fig. 5 , section 2). 59.9% were told by their physicians that there was no treatment option available ( Fig. 5 , section 3). We saw no clear differences between WHO regions.

Concerning treatment, 21.5% (222) reported non-surgical approaches to cure floaters. Most common answers were Atropine and eye drops in general, antioxidants, different vitamins and supplements, fish oil, bromelain, sunglasses, drinking enough water, meditation, and traditional Chinese medicine. Vitrocap N capsules for dietary management of vitreous degeneration of the eyes were also often mentioned. No participant however asserted to have discovered a cure for themselves.

About 54.6% of the total of 110 individuals with LV were not satisfied with the outcome, with 68.1% reporting that their floater symptoms had remained the same or worsened after LV. Slightly higher satisfaction was reported by 86 attendees on PPV ( Fig. 5 , section 4, section 5). Comparable numbers of participants reported either posttreatment recurrence or improvement for both treatments ( Fig. 5 , section 6). The major complication within one year was PVD (LV 14.5%; PPV 29.1%). 61.8% stated no complications connected with LV compared to 38.4% for PPV ( Fig. 5 , section 7).

Of the 438 individuals who took no action regarding their floaters, 38.6% stated that they could not find a doctor offering a suitable treatment, 48.4% pointed to the high risks of PPV and LV, 8.0% stated high treatment costs as a factor, and 26.7% had been advised against treatment.

Additional floaters after certain procedures mainly appeared in cataract, retinal, and refractive-laser surgery ( Fig. 5 , section 8).

### Refractive error

**Fig. 6:**
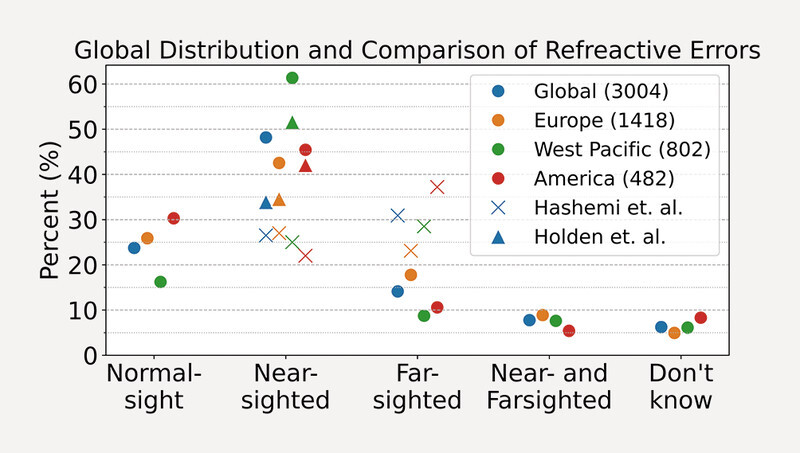
Hashemi et al. 30 consistently reported fewer near- and more far-sighted individuals than we observed in our dataset. The same is true for Holden et al. 31 considering the individual world regions. Both reviews contain fewer short-sighted people than our dataset of participants affected by floaters.

A total of 3004 eyes are included in the dataset. When compared to the global distribution of myopia and hyperopia reported in 30 , 31 , we observed a higher prevalence of myopia and a lower prevalence of hyperopia in our dataset ( Fig. 6 ).

A similar floater survey conducted on 603 smartphone users observed 3.5 times more myopes and 4.4 times more hyperopes than emmetropes with floaters 8 . We observed the opposite for hyperopes. It should be noted that 85% of participants in the West Pacific data come from China, which is generally facing a high prevalence of rising myopia figures 32 .

No correlation between the refractive error and the number of floaters selected in the sketching tool was observed.

## Discussion

To the best of our knowledge, we have collected the largest dataset on people affected by floaters to date. We presented a new tool to recreate a personal field of view with floaters, allowing us to reveal different prevalences of floater types over age. Furthermore, with 41% of participants in their twenties, our data shows that young people can also suffer from floaters and their experiences should be considered as valid as those of older patients.

### Bias

Since this survey was available online to everyone, the study population must be assumed to be biased. People with negative experiences and severe symptoms are more likely to actively engage with the topic, and the online format was possibly more appealing to a younger population. These considerations must be kept in mind when drawing conclusions about a more general population.

### Personal experience and quality of life

The survey clearly showed that a high proportion of participants significantly suffer from experiencing floaters. Floaters can have a high psychological burden. The present and previous studies strongly highlight the importance of awareness both in the general public and among physicians 9 , 12 , 14 , 15 , 16 .

### Floater sketcher

The tool attempted to quantify the objective level of suffering experienced by individuals. As such, it could assist doctors in better assessing the patientʼs level of suffering. However, SSI and OSI showed just a weak correlation in our study. For a large percentage of participants, the difference between the two indices is less than 5%, demonstrating the potential of measuring suffering from SVO with a standardized tool. A frequent feedback was the lack of consideration of the movement of the floaters in the sketcher. This was not implemented for reasons of technical feasibility and ease of use and should be integrated in a future version.

A questionnaire designed specifically to match floater properties might make a more robust correlation possible, but since such questioning would be highly specific and time consuming, we opted for broader data collection instead. The tool can be of additional use in the context of patient examination, and will be made available by Laser Zentrum Hannover e.V. via the following link: https://www.lzh.de/en/form/floater-study-sketching-tool .

Our results highlight the highly subjective nature of individual suffering. The floater sketcher may a useful communication aid for patients and physicians. Harmer et al. demonstrated that floaters close to the retina produce a stronger shadow, while those further away appear larger but more blurred 11 . Using the DoC and BT parameters, it might be possible to better locate the floater in space.

The decrease in translucent floaters with age ( Fig. 4 ) might be explained by translucent tubular structures being remnants of embryonic hyaloid vasculature, while darker dots and strings consist of collagen aggregates 5 . Nevertheless, we observed dot-like and string-like floaters across all age groups, suggesting that collagen aggregates are not exclusive to the elderly population.

### Treatment

The apparent dissatisfaction of affected individuals with currently available patient care underscores the need and economic potential for the development of suitable treatment options. A large proportion of participants in our study were told by their physicians that no treatment was available, and left without advice on managing their symptoms. Moreover, only 13.1% were told how to manage their symptoms. An always possible advice is to minimize the amount of incident light, for example by wearing sunglasses or using the dark mode in computer software. While symptomatic floaters often disappear within months, individuals with persistent SVO tend to be overlooked, as is evidenced by the large proportion of people in our dataset who have been suffering for years and feel strongly affected during everyday life.

Both PPV and LV are associated with considerable risks 20 , 23 , 25 , 26 , 33 , 34 , 35 , 36 . Furthermore, our data indicates a relatively low satisfaction rate, although the bias in the study population must be considered here. The complication rates associated with PPV also differ from those published before 20 , 37 . In accordance with the published studies however, our results strongly underline the need for safer and more effective treatment options.

Despite the data suggesting a trend towards a higher satisfaction rate with vitrectomy for floaters, a higher number of complications were also reported with vitrectomy one year after surgery, with only 38.4% having no complications.

In comparison with the literature, these are low success rates and high incidences of complication. With approximately every third patient affected, cataract is the most commonly reported complication of PPV in literature 20 , 21 , 36 , but only 10.5% (10) of our participants experienced cataract. However, the complication rates of PVD (29.1%, 25 individuals) and eye infection (26.7%, 23 individuals) are markedly higher. These high numbers stand in contradiction with the current literature 38 , 39 , 40 . The majority of our participants are young (mean age 35), indicating that the vitreous body has not yet detached. This is arguably the most challenging group to manage. In contrast, the average age of participants in other intervention studies is usually around 60 years 20 , 41 , 42 , 43 . In addition, we point out that the data was provided by non-experts and that the reported complication rates in particular might not reflect the clinical reality 20 , 33 .

Regarding the treatment, we suspect to have recruited more people with negative experiences, which can be linked to treatment failure or unrealistic patient expectations.

Additional floaters after different ophthalmological interventions most often appeared after surgeries that restore the visual quality, e.g. cataract and refractive laser surgery. This does not necessarily mean that new floaters have appeared during these procedures, but perhaps that they are more noticeable to the patient after treatment due to the changed light exposure.

### Refractive error

The results clearly show a high number of myopic attendees. Myopia related floaters are known to appear in younger patients. Considering the globally increasing prevalence of myopia, we can expect an increase in patients with symptomatic vitreous opacities as well.

## Conclusion

Here we presented the data from an online survey of a large group of participants. Even if the data is biased, it gives an insight into possible fields in which a clinical trial is worthwhile. It highlights the need for improved awareness and patient care. In this sense, the Floater Sketcher can facilitate communication between patients and physicians.

Conclusion Box **Already known:**

- The morphologies of vitreous floaters are rarely differentiated in the literature.
- Individual assessment of severity remains a difficult task.
- The correlation between myopia and vitreous opacities is widely recognized, but it has not been compared against global data on refractive status.

**Newly described:**

- This work proposes a new method to measure individual suffering using the reconstruction of the personal visual field with floaters.
- Floater morphology appears to be age dependent. Future and current floater type dependent treatments are therefore targeting a different generation of patients.
- Compared to global public data, our dataset of participants affected by floaters shows a higher prevalence of myopia and a lower prevalence of hyperopia. As myopia increases in the global population, the number of people with symptomatic vitreous opacities is also likely to increase.

## Declarations

Ethical approval and consent to participate. This study adhered to the ethical principles of the Declaration of Helsinki. Since all data were fully anonymized and no identifiable information was collected, ethical approval was not required. The research complied with the EU General Data Protection Regulation (GDPR), and participation was entirely voluntary. The study design was considered ethically uncritical according to the standards of the German Research Foundation (DFG).

Consent for publication: not applicable.

## Supporting Information

Ergänzendes Material Supporting Information
