# Supplementary material for: Investigating Symptomatic Vitreous Opacities: An Online Survey and Field of View Reconstruction
Source: Klin Monbl Augenheilkd. 2025 Oct 15;242(10):991–1000. doi: 10.1055/a-2676-7455 (PMC12527563; doi:10.1055/a-2676-7455)
Supplement: Supplementary file 1 — Ergänzendes Material [file 10-1055-a-2676-7455-sup_kl3262.pdf]

Supplementary material

Table S1 – Subjective Suffering Index, questions and scoring

1 When you notice your floaters, for how long do they bother you?

Not at all	A few seconds	A few minutes	Up to one hour	Several hours	Several days
0	1	2	3	4	5

2How much did the floaters bother you in the past four weeks?

Not at all	Barely	A little	More severely	Strongly
0	1	2	3	4

3 How strongly have the floaters affected you during the past four weeks because you could not identify something (e.g. pictures) or could not read writing?

Not at all	Barely	A little	More severely	Strongly
0	1	2	3	4

4 In which situations did you feel particularly affected?

	Does not apply at all	Does generally not apply	Partly applies	Generally applies	Absolutely applies
Reading small writing	0	1	2	3	4
Reading in general	0	1	2	3	4
Working at a monitor	0	1	2	3	4
Driving during the day	0	1	2	3	4
Driving during the night	0	1	2	3	4
Outdoor activities	0	1	2	3	4
**5 Please indicate whether the following statements apply to you: Because of my floaters,…**					
I have stopped going to the movies, theater or similar events.	0	1	2	3	4
I no longer drive during the day.	0	1	2	3	4
I no longer drive during the night.	0	1	2	3	4
I read less in general.	0	1	2	3	4
**6 Please indicate whether the following statements apply to you: My floaters…**					
Are frequently not taken seriously.	0	1	2	3	4
Cause situations I find embarrassing.	0	1	2	3	4
Cause me to be less productive at work.	0	1	2	3	4
Hinder me from pursuing hobbies or participating in activities.	0	1	2	3	4

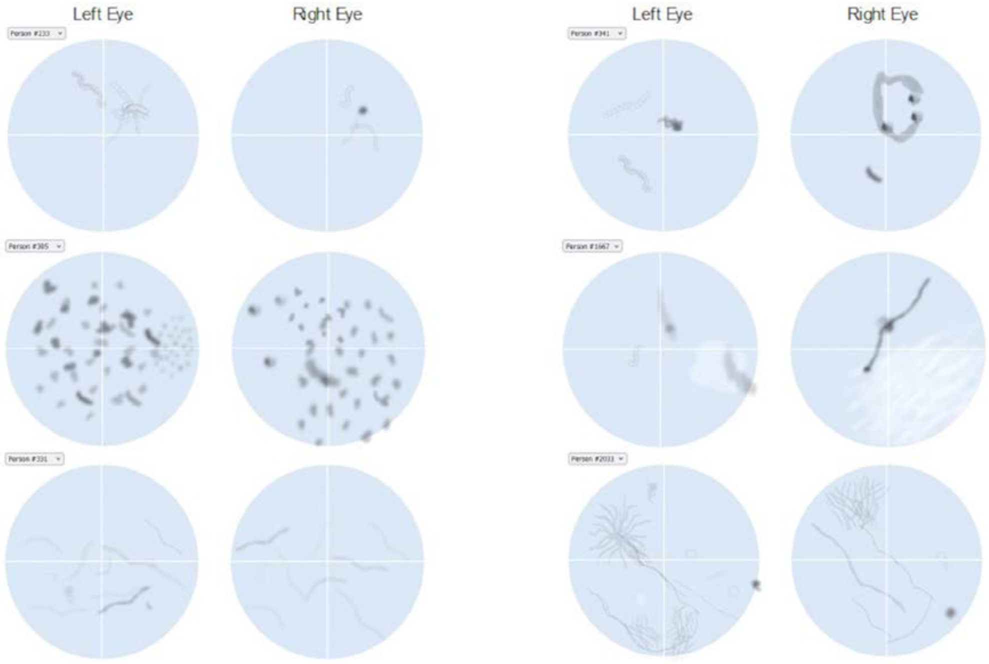

Fig. S1. Six representative examples of participants' field-of-view reconstructions based on survey data.
